# Association between serum albumin-to-creatinine ratio and clinical outcomes among patients with ST-elevation myocardial infarction after percutaneous coronary intervention: a secondary analysis based on Dryad databases

**DOI:** 10.3389/fcvm.2023.1191167

**Published:** 2023-06-29

**Authors:** Xiaoye Huang, Yuchun Liu, Chuyang Zhong, Zengrui Lin, Binyun Zheng

**Affiliations:** ^1^ Intensive Care Unit, Jieyang People’s Hospital, Jieyang, China; ^2^ Emergency Department, Jieyang People’s Hospital, Jieyang, China

**Keywords:** serum albumin, creatinine, ST-elevation myocardial infarction, percutaneous coronary intervention, clinical outcomes

## Abstract

**Background:**

The prognostic value of the serum albumin-to-creatinine ratio (sACR) in patients with ST-elevation myocardial infarction (STEMI) remains unclear. This study aims to investigate the impact of the sACR on incident major adverse cardiovascular events (MACEs) among revascularized patients with STEMI at long-term follow-up.

**Methods:**

A total of 461 patients with STEMI who underwent successful primary percutaneous coronary intervention (PCI) were enrolled to explore the association between the sACR and MACE during a 30-month follow-up. The Cox regression proportional hazard model was used to evaluate the prognostic value of the sACR. Heterogeneity among specific groups was investigated by subgroup analysis.

**Results:**

A total of 118 patients developed MACE during the follow-up. A negative association between the sACR and MACE was found after adjusting for other MACE-related risk factors. In subgroup analyses, the sACR was inversely associated with MACE in patients aged ≥ 60 years [hazard ratio (HR), 0.478; 95% confidence interval (CI), 0.292–0.784], male (HR, 0.528; 95% CI, 0.327–0.851), with hypertension history (HR, 0.470; 95% CI, 0.271–0.816), and with anterior wall myocardial infarction (HR, 0.418; 95% CI, 0.239–0.730). Meanwhile, the negative association between the sACR and MACE remained significant in a sensitivity analysis that excluded patients with low serum albumin levels (HR, 0.553; 95% CI, 0.356–0.860).

**Conclusions:**

Patients with STEMI who underwent successful PCI with a low sACR had a higher risk of developing MACE, indicating that the sACR could be used to identify patients with STEMI who are at high risk of developing MACE.

## 1. Introduction

ST-segment elevation myocardial infarction (STEMI) is the most severe and time-sensitive disease with high mortality among patients with coronary artery disease (1). Worldwide, STEMI is the leading cause of death, which results in a high medical burden (2). Recent years have witnessed great advances in the treatment of acute myocardial infarction (AMI), but the risk of death remains high in patients with STEMI (2, 3). The decreasing trend in STEMI mortality is mainly attributed to the greater use of reperfusion therapy, especially primary percutaneous coronary intervention (PCI) (3, 4). Actually, the prognosis of post-STEMI following PCI is influenced by clinical, echocardiographic, and biochemical factors (5). Therefore, early risk stratification is extremely essential for clinicians to determine treatment strategy and long-term management.

Inflammation and platelet activation play an important role in the pathogenesis of AMI (6). Serum albumin, a stabilizing protein correlated with inflammation and platelet activation, has been found to be an essential biomarker for adverse events of AMI (7–9). Meanwhile, serum creatinine is an indicator of renal function and is associated with oxidative stress, endothelial function, inflammation, and systemic atherosclerosis (10, 11). The elevated serum creatinine levels at admission could also help in the prediction of the onset of adverse events in patients with AMI (11–14). Compared with serum albumin or creatinine alone, the serum albumin to creatinine ratio (sACR) has been proven to be a more valuable biomarker for adverse events in patients with AMI (15, 16). Unfortunately, whether the sACR is associated with the adverse events of STEMI after successful primary PCI remains unknown. More importantly, the clinical application of the sACR may be complicated by different age, gender, and clinical conditions. Hence, this study aims to investigate the impact of the sACR on incident major adverse cardiovascular events (MACEs) among revascularized patients with STEMI at long-term follow-up.

## 2. Materials and methods

### 2.1. Data source and study population

The data used in this present study were collected from the Dryad Digital Repository (https://datadryad.org/), which houses a large number of datasets from previously published papers (17). The original study was conducted in the First People's Hospital of Taizhou to explore the association of apelin-12 with MACE after primary PCI in patients with STEMI (18). A total of 464 consecutive patients with STEMI meeting the diagnostic criteria for STEMI between January 2010 and October 2014 were included in the original study. The diagnostic criteria of STEMI were typical persistent chest pain lasting more than 30 min, prolonged electrocardiograph alteration, and significantly elevated serum myocardial enzyme and troponin levels. All included patients had received successful standard primary PCI. The exclusion criteria and detailed treatment have been described previously (18). In addition, two patients with serum creatinine levels of more than 133 mmol/L and one patient with missing serum albumin were excluded, and the remaining 461 patients were included in the final analysis. All participants signed the informed consent form. Ethical approval was no longer required for this study because all data were anonymized, and data analysis adhered to the rules of the Dryad Digital Repository.

### 2.2. Data collection

The following basic characteristics of all patients were collected at hospital admission: age, gender, history of hypertension, history of diabetes mellitus, history of myocardial infarction, systolic blood pressure, heart rate, Killip's grade, and anterior wall myocardial infarction. The following laboratory test parameters were determined within 48 h of admission to hospital: white blood cell (WBC), percentage of neutrophils, hemoglobin, platelets, albumin, urea nitrogen, fasting blood glucose (FBG), uric acid, total cholesterol (TC), triglyceride (TG), high-density lipoprotein (HDL-C), low-density lipoprotein-cholesterol (LDL), peak cardiac troponin I (cTnI), and peak creatine kinase-MB (CK-MB). Echocardiographic and electrocardiographic data such as left atrial diameter, left ventricular diastolic diameter (LVEDD), and pathological Q wave were collected. Stent number, culprit vessel, and Gensini score were determined by interventional surgeons.

### 2.3. Definitions

The sACR is defined as the serum albumin divided by the serum creatinine. All patients were followed up for 30 months after primary PCI or until the occurrence of MACE. MACE is a combination of clinically driven target lesion revascularization, recurrent target vessel myocardial infarction, cardiogenic shock, congestive heart failure, or cardiac death.

### 2.4. Statistical analysis

Patients were divided into two groups according to the median of the sACR (0.513): high (>0.513) and low (≤0.513) levels. Continuous variables were expressed as mean ± standard deviation (mean ± SD) for normally distributed data or median (25th and 75th percentiles) for non-normally distributed data, while categorical variables were presented as numbers and percentages. Continuous variables were compared by using the Mann–Whitney *U* test or Student’s *t*-test as appropriate, and categorical variables were analyzed by using the chi-square test. A Kaplan–Meier analysis was performed to acquire a graphical presentation of time to the incidence of MACE, and the log-rank test was used to compare the difference between the two groups according to the sACR. The hazard ratio (HR) and the 95% confidence interval (CI) were calculated to explore the association between the sACR and the incidence of MACE using Cox proportional hazard models. All predictors were included in the multiple Cox regression models based on their clinical importance. Model 1 was the crude model with no variables adjusted. Model 2 was adjusted for age, gender, history of hypotension, history of diabetes, history of myocardial infarction, pathological Q wave, Killip's grade, systolic blood pressure, and heart rate. Model 3 was based on Model 2 and all other covariates. Then, subgroup analyses were performed according to age, gender, history of hypertension, history of diabetes mellitus, pathological Q wave, and anterior wall myocardial infarction. All statistical analyses were performed using the R package (version 3.4.3). A *P*-value < 0.05 (two-sided) was considered statistically significant.

## 3. Results

**Figure 1:**
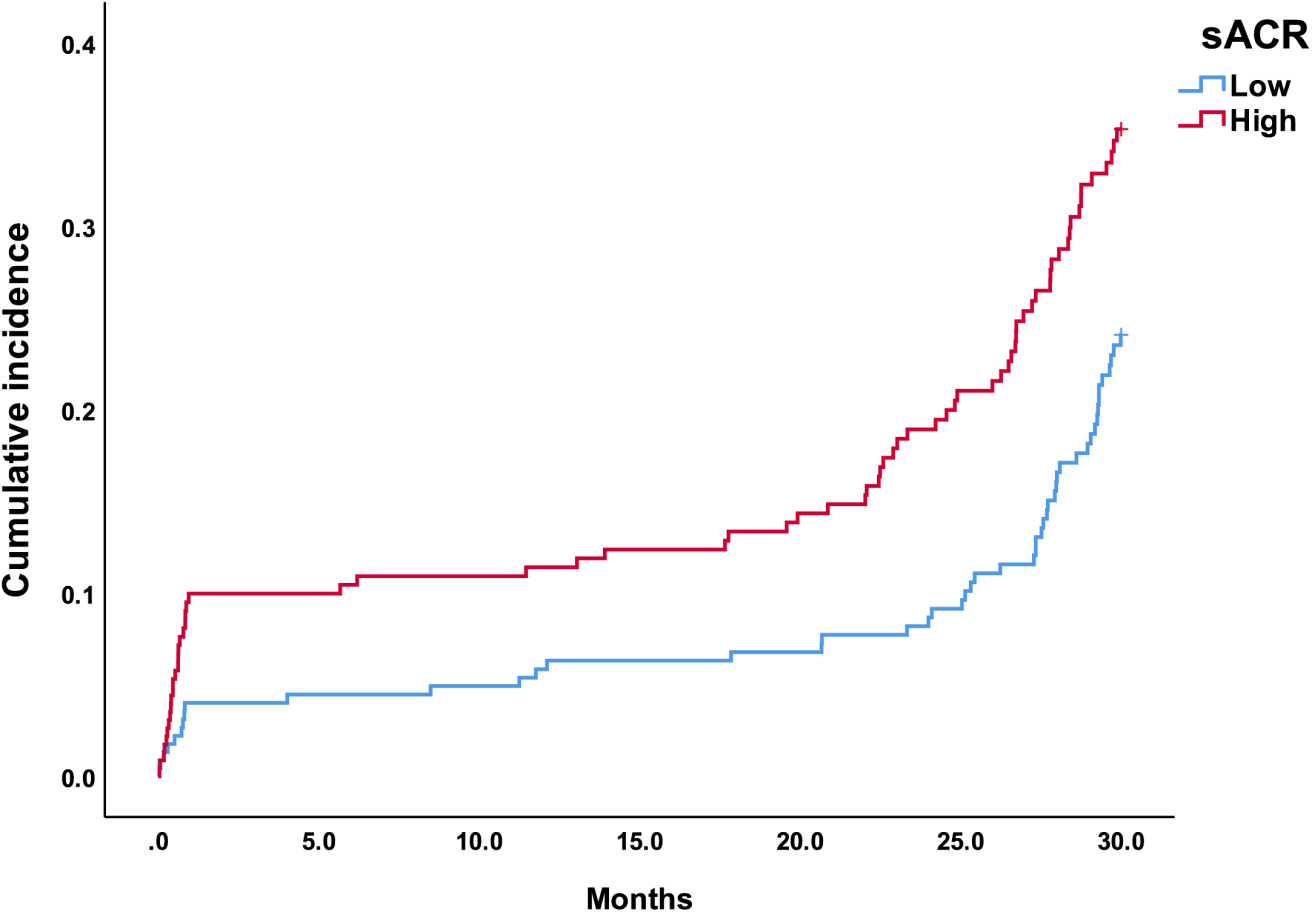
Kaplan–Meier curves stratified by the sACR. The curves show different incidences of MACE in patients with STEMI with different sACRs.

The baseline characteristics of the enrolled patients are summarized in Table 1, according to the median of the sACR. Two hundred and thirty-two patients with an sACR ≤ 0.513 were assigned to the low sACR group, while the remaining patients with an sACR ≤ 0.513 were assigned to the high sACR group. No significant differences were found in most of the analyzed variables between the high sACR group and the low sACR group, except in terms of age, WBC count, percentage of neutrophils, albumin, creatinine, LDL, culprit vessels, and MACE occurrence. Figure 1 shows Kaplan–Meier analysis illustrating the cumulative incidence of MACE stratified by the sACR. Patients with a lower sACR showed a higher risk of MACE compared with those with a higher sACR (*P* = 0.026).

**Table 1:** Relationships between clinical and laboratory data and the sACR in patients with ST-elevation myocardial infarction.

Variables	Total (*N* = 461)	sACR		*p*-Value
		(High, >0.513, *N* = 229)	(Low, ≤0.513, *N* = 232)	
Age (years)	63 ± 11.9	61 ± 11.3	65 ± 12.2	0.003
Gender (*n*, male/female)	355/106	171/58	184/48	0.237
Hypotension history (*n*, yes/no)	263/198	137/92	126/106	0.232
Diabetes history (*n*, yes/no)	148/313	78/151	70/162	0.371
Myocardial infarction history (*n*, yes/no)	54/407	30/199	24/208	0.358
SBP (mm Hg)	131 (109–153)	129 (106–154.5)	132.5 (112.25–152)	0.279
Heart rate (beats/min)	77 ± 17	76 ± 16	78 ± 18	0.374
Pathological Q wave (*n*, yes/no)	222/239	111/118	111/121	0.893
AWMI (*n*, yes/no)	230/231	110/119	120/112	0.428
Killip's grade (*n*, ≥II/I)	110/351	51/178	59/173	0.426
Stent number (*n*, ≥II/I)	160/301	82/147	78/158	0.622
LVEDD (mm)	50 (45–56)	50 (45–56)	50 (45–56)	0.592
left atrial diameter (mm)	38 (33–42)	38 (33–42)	38 (33–41)	0.871
WBC (×10^9^/l)	9.96 (7.16–12.96)	10.24 (7.69–13.29)	9.16 (6.96–11.96)	0.05
Neutrophils (%)	77.6 (66–85.1)	74.8 (64.8–84.7)	78.9 (68.9–85.8)	0.016
Hemoglobin (g/l)	144 (131–158)	144.5 (133–156)	143 (131–159)	0.845
Platelet (×10^9^/l)	231 (183–272)	229.5 (183–272)	241 (182–281)	0.581
Albumin (g/l)	38 (35–41)	39.7 (37–42)	37 (34–40)	<0.001
Urea nitrogen	6.72 ± 2.07	6.69 ± 2.08	6.76 ± 2.06	0.718
FBG (mmol/l)	7.12 (5.79–9.71)	7.41 (5.93–9.70)	6.94 (5.60–9.72)	0.316
Creatinine (μmol/l)	75 (61.6–85.6)	61.2 (53–70.1)	85.3 (78–91)	<0.001
Uric acid (μmol/l)	335 (282–390)	330 (270–389)	350 (290–390)	0.064
TC (mmol/l)	5.66 ± 1.11	5.62 ± 1.14	5.69 ± 1.07	0.489
TG (mmol/l)	0.97 (0.54–1.54)	0.97 (0.57–1.54)	0.98 (0.51–1.54)	0.921
HDL-C (mmol/l)	1.21 (1–1.39)	1.22 (1–1.40)	1.19 (0.98–1.40)	0.834
LDL (mmol/l)	3 (2.48–3.60)	3.1 (2.5–3.72)	2.9 (2.32–3.46)	0.006
Peak cTnI (ng/ml)	13.6 (4.32–29)	13.4 (3.91–27.7	11.1 (4.2–28.3)	0.922
Peak CK-MB (U/l)	106 (45.5–195.5)	101 (39–192.5)	103 (40.5–185)	0.118
Gensini score	74 (43–101)	73 (44–107)	72 (34–99)	0.361
Culprit vessels				
LAD (*n*, %)	232 (50.3)	104 (45.4)	128 (55.2)	0.018
LCX (*n*, %)	72 (15.6)	46 (20.1)	26 (11.2)	
RCA (*n*, %)	157 (34.1)	79 (34.5)	78(33.6)	
MACEs (*n*, %)	118(25.6)	49(21.4)	69(29.7)	0.04

sACR, serum albumin-to-serum creatinine ratio; SBP, systolic blood pressure; AWMI, anterior wall myocardial infarction; LVEDD, left ventricular and diastolic diameter; WBC, white blood cells; FBG, fasting blood glucose; TC, total cholesterol; TG, triglyceride; HDL-C, high-density lipoprotein; LDL-C, low-density lipoprotein-cholesterol; cTnI, cardiac troponin I; CK-MB, creatine kinase-MB; LAD, left anterior descending coronary artery; LCX, left circumflex coronary artery; RCA, right coronary artery; MACEs, major adverse coronary events.

The results of the Cox hazard model of the sACR on incident MACE are presented in Table 2. In Model 1, the sACR was negatively associated with MACE (HR, 0.661; 95% CI, 0.458–0.953). After adjustment for confounding factors, the negative association still remained in Model 2 (HR, 0.687; 95% CI, 0.473–0.998) and Model 3 (HR, 0.637; 95% CI, 0.410–0.989).

**Table 2:** Cox regression analysis for determining the relationship between sACR and MACE.

sACR (low vs. high)	HR (95% CI)	*p*-Value
Model 1	0.661 (0.458–0.953)	0.027
Model 2	0.687 (0.473–0.998)	0.049
Model 3	0.634 (0.428–0.938)	0.023

Model 1 was a crude model with no variables adjusted.

Model 2 adjusted for age, gender, hypotension history, diabetes history, myocardial infarction history, pathological Q wave, Killip's grade, systolic blood pressure, and heart rate.

Model 3 adjusted for age, gender, hypotension history, diabetes history, myocardial infarction history, pathological Q wave, Killip's grade, systolic blood pressure, heart rate, culprit vessels, anterior wall myocardial infarction, stent number, left ventricular and diastolic diameter, left atrial diameter, Gensini score, white blood cells, percentage of neutrophils, hemoglobin, platelets, urea nitrogen, fasting blood glucose, uric acid, total cholesterol, triglyceride, high-density lipoprotein, low-density lipoprotein-cholesterol, peak cardiac troponin I, and peak creatine kinase-MB.

**Figure 2:**
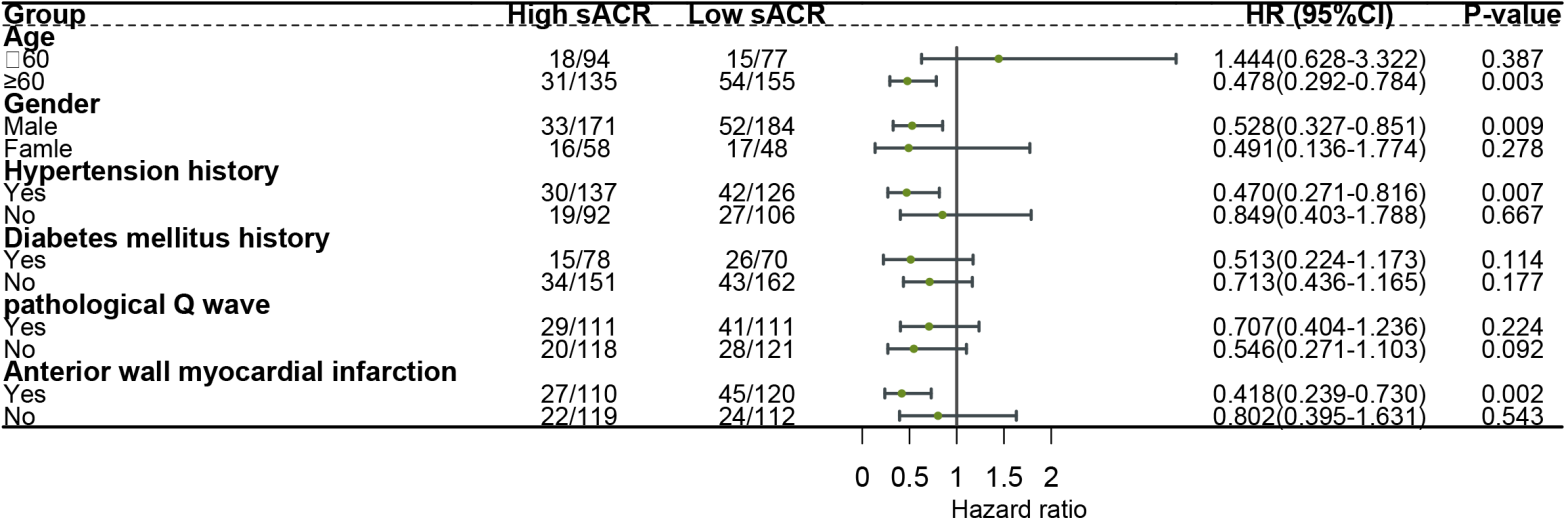
Effect size of the sACR on incident MACE in different subgroups. Each stratification was adjusted for age, gender, hypotension history, diabetes history, myocardial infarction history, pathological Q wave, Killip's grade, systolic blood pressure, heart rate, culprit vessels, anterior wall myocardial infarction, stent number, left ventricular and diastolic diameter, left atrial diameter, Gensini score, white blood cells, percentage of neutrophils, hemoglobin, platelets, urea nitrogen, fasting blood glucose, uric acid, total cholesterol, triglyceride, high-density lipoprotein, low-density lipoprotein-cholesterol, peak cardiac troponin I, and peak creatine kinase-MB, except the stratification factor itself.

The sACR was inversely associated with MACE among participants aged ≥ 60 years (HR, 0.478; 95% CI, 0.292–0.784), males (HR, 0.528 95% CI, 0.327–0.851), with a history of hypertension (HR, 0.470; 95% CI, 0.271–0.816), and with anterior wall myocardial infarction (HR, 0.418; 95% CI, 0.239–0.730) (Figure 2). After excluding patients with low serum albumin levels (more than 35 g/L), the sACR was still inversely associated with MACE among patients with STEMI (HR, 0.553; 95% CI, 0.356–0.860).

## 4. Discussion

In this cohort study, the sACR was found to be inversely associated with MACE in patients with STEMI after primary PCI. After excluding patients with low serum albumin levels, this inverse association remained. A subgroup analysis indicated that a low sACR was a risk factor for STEMI patients aged ≥ 60 years, male, with hypertension history, and with anterior wall myocardial infarction.

The sACR is a reproducible, low-cost parameter that is easy to collect in routine clinical management. Therefore, this investigation enriches the recent evidence that a low sACR could be used for predicting the occurrence of adverse events among patients with AMI (15, 16). In China, only one study that included 2,250 patients with a 20-month follow-up reported that a low sACR is associated with an increased risk of all-cause mortality among patients with AMI after PCI (15). The study by Turkyilmaz et al. showed that the sACR was an independent predictor of in-hospital mortality, as well as contrast-induced nephropathy, congestive heart failure, and stent thrombosis at 30 days among patients with STEMI (16). However, no research has been done to investigate the link between the sACR and the risk of MACE during long-term follow-up among patients with STEMI after primary PCI. Hence, this study was the first evaluation of the predictive potential of the sACR for MACE among revascularized patients with STEMI at long-term follow-up (30 months).

The exact mechanisms underlying the association of the sACR and the risk of MACE among revascularized patients with STEMI have still not been fully understood. Inflammation and thrombosis play important pathological roles in STEMI (6). Decreased serum albumin is associated with an increased risk of adverse events in patients with AMI (7, 9). For each 1 g/dl reduction in serum albumin levels in patients with first-onset AMI, the risk of all-cause mortality and cardiovascular death increased by 66% and 47%, respectively (8). Inflammation not only reduces serum albumin synthesis but also increases serum albumin catabolism (19). Physiological concentrations of serum albumin would exert its anti-inflammatory effect on endothelial cells (20). Moreover, decreased serum albumin has been shown to be related to the promotion of oxidative stress, platelet activation, and aggregation, which further increase the risk of thrombotic events (7, 9, 21). Creatinine is an indicator of kidney function and an important marker of oxidative stress, endothelial dysfunction, inflammatory status, and progressive atherosclerosis (10, 11, 14). The elevated serum creatinine levels at admission have an independent ability to predict adverse events in patients with AMI (11–14). However, it must be noted that patients with serum creatinine levels of more than 133 mmol/L were excluded from this study. Increased serum creatinine levels within the normal range can increase the risk of cardiovascular disease among participants without metabolic syndrome (22). Increased serum creatinine levels within the normal range could increase the risk of cardiovascular disease by mediating an inflammatory state augmented by dysfunctional apolipoprotein A-I (22). Therefore, combining serum albumin and creatinine in patients with STEMI suffering from an acute inflammatory and thrombotic process may help effectively identify patients with a high risk of adverse events.

The study by Plakht et al. found that decreased serum albumin within the normal clinical range also increases the long-term all-cause mortality in hospital survivors after AMI (7). After excluding patients with low serum albumin levels, the inverse association between the sACR and the risk of MACE among revascularized patients with STEMI remained significant. Therefore, it is necessary to calculate the sACR to assess the risk of MACE among patients with STEMI with normal serum albumin levels.

Previous studies have not evaluated the relationship between the sACR and the risk of MACE in different subgroups. Our subgroup analysis demonstrated that a low sACR was a risk factor for patients aged ≥60 years, male, with hypertension history, and with anterior wall myocardial infarction. Owing to the limited number of patients included in the present study, this result should be interpreted with caution. More importantly, more prospective studies are needed in this area.

Several limitations can be found in this study. First, unmeasured confounding factors such as smoking, high-sensitivity C-reactive protein, and the use of statins or beta blockers and antiplatelet drugs, may be attributed to the occurrence of MACE. This study was a secondary analysis based on previous studies, and related information about other confounders was limited. Second, repeated evaluations of serum creatinine levels during hospitalization can help improve risk assessment among patients with AMI (23), but the sACR was measured only once on admission. The variations in the sACR during hospitalization and its predictive value of adverse events were not clarified in this study. Third, we excluded patients with serum creatinine levels exceeding 133 mmol/L. However, chronic kidney disease and atherosclerotic cardiovascular disease often coexist. A significant proportion of patients with STEMI present with serum creatinine levels higher than 133 mmol/L. Therefore, the exclusion of these patients may affect the results of the study. Finally, this study had a relatively small sample size, especially when subgroup analysis was performed. Therefore, further research is needed to generalize our conclusions.

## 5. Conclusion

A low sACR was associated with an increased risk of MACE in patients with STEMI after primary PCI. Screening the sACR of individuals could be used to stratify patients with STEMI at risk of MACE.

## Data Availability

The datasets presented in this study can be found in online repositories. The names of the repository/repositories and accession number(s) can be found below: https://datadryad.org/stash/dataset/doi:10.5061%2Fdryad.pf56m.
